# Supplementary material for: VBASS enables integration of single cell gene expression data in Bayesian association analysis of rare variants
Source: Commun Biol. 2023 Jul 25;6:774. doi: 10.1038/s42003-023-05155-9 (PMC10368729; doi:10.1038/s42003-023-05155-9)
Supplement: Supplementary file 2 — Supplementary Information [file 42003_2023_5155_MOESM2_ESM.pdf]

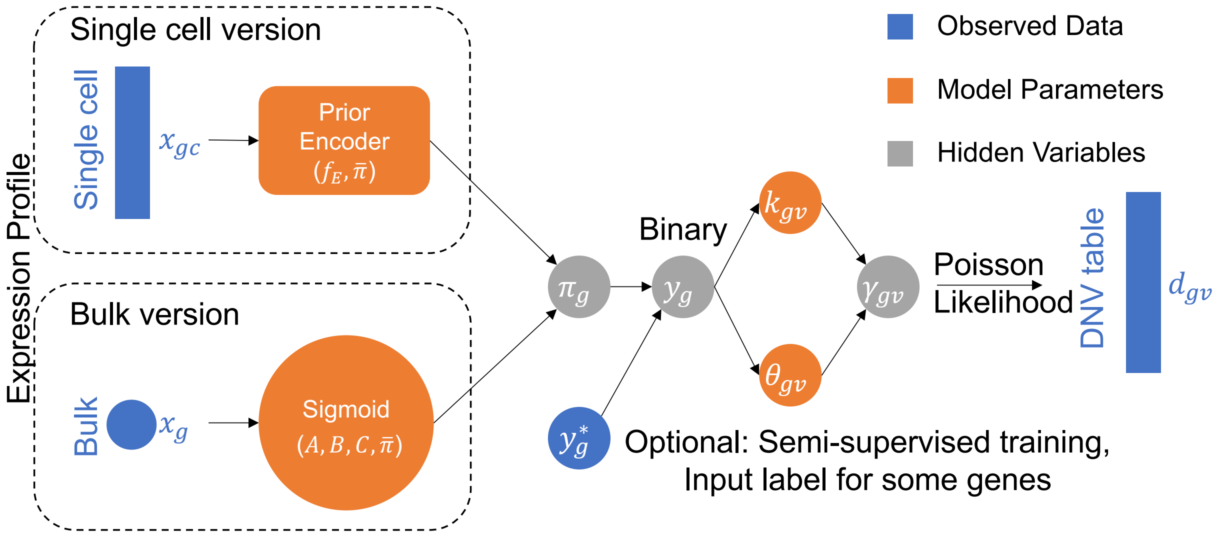

Supplementary Figure 1. Graphic model structure of VBASS. Nodes represent random variables or parameters, and arrows represent dependency. The input could be either a vector of single cell expression profile or a scalar of bulk expression profile. For the single cell version with vector input, f_E is a neural network to inference the gene specific parameter π_g with KL penalty parameterized by $\bar{\pi}$, while for the bulk version with scalar input, it could be simplified to a sigmoid function with four parameters, $A, B, C, \bar{\pi}$. π_g will parameterize a Bernoulli distributions of y_g. k_{gv}, θ_{gv} are two random variables conditioned on y_g that reconstruct the parameters of Gamma-Poisson distribution for d_{gv}.

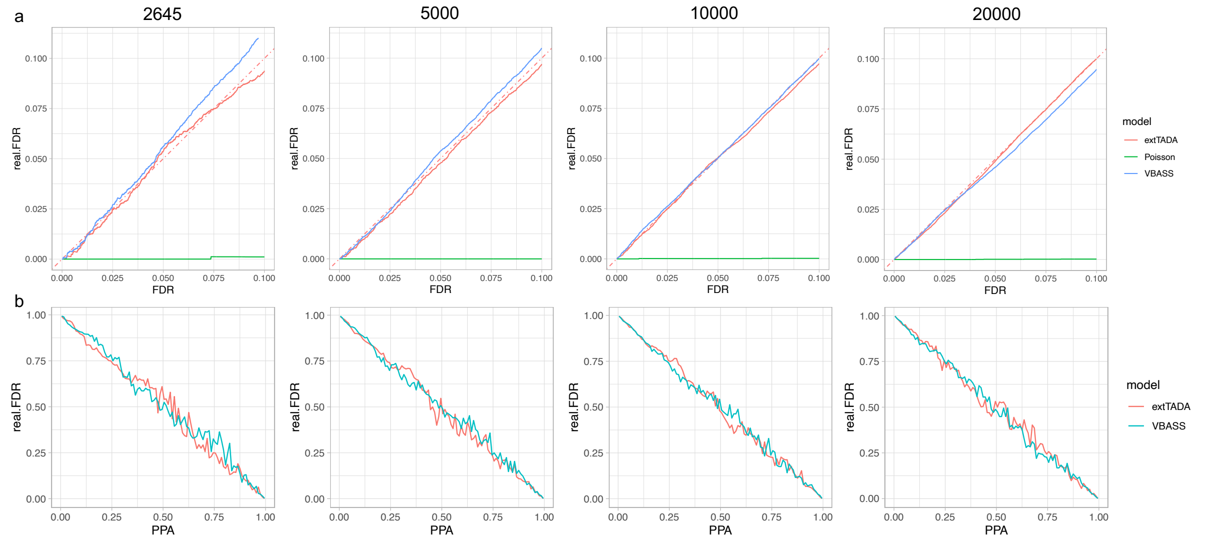

Supplementary Figure 2. Local and global false discovery rate control of VBASS. a) False discovery control for two models and Poisson test, only show the part with FDR ≤ 0.1. X-axis, estimated false discovery rate (FDR) from the model, y-axis, real false discovery rate in simulation. b) Local false discovery control for two models. X-axis, posterior probability attribute (PPA) from the two model, y-axis, real false discovery rate in simulation. Each dot represents a gene set with 100 genes with close PPA.

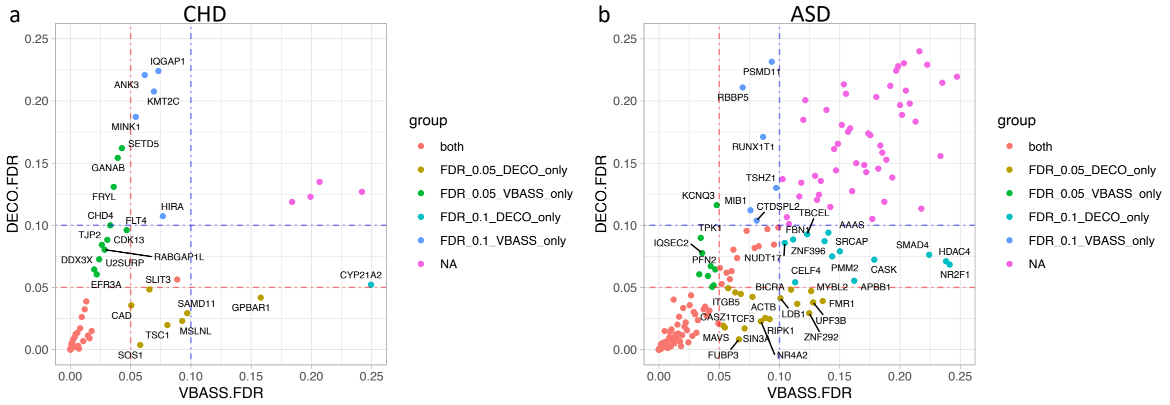

Supplementary Figure 3. Comparison of VBASS and DECO on CHD and ASD de novo variants dataset. Genes were colored by the significance in both models or only in one model at FDR ≤ 0.05 and FDR ≤ 0.1. a) CHD. b) ASD

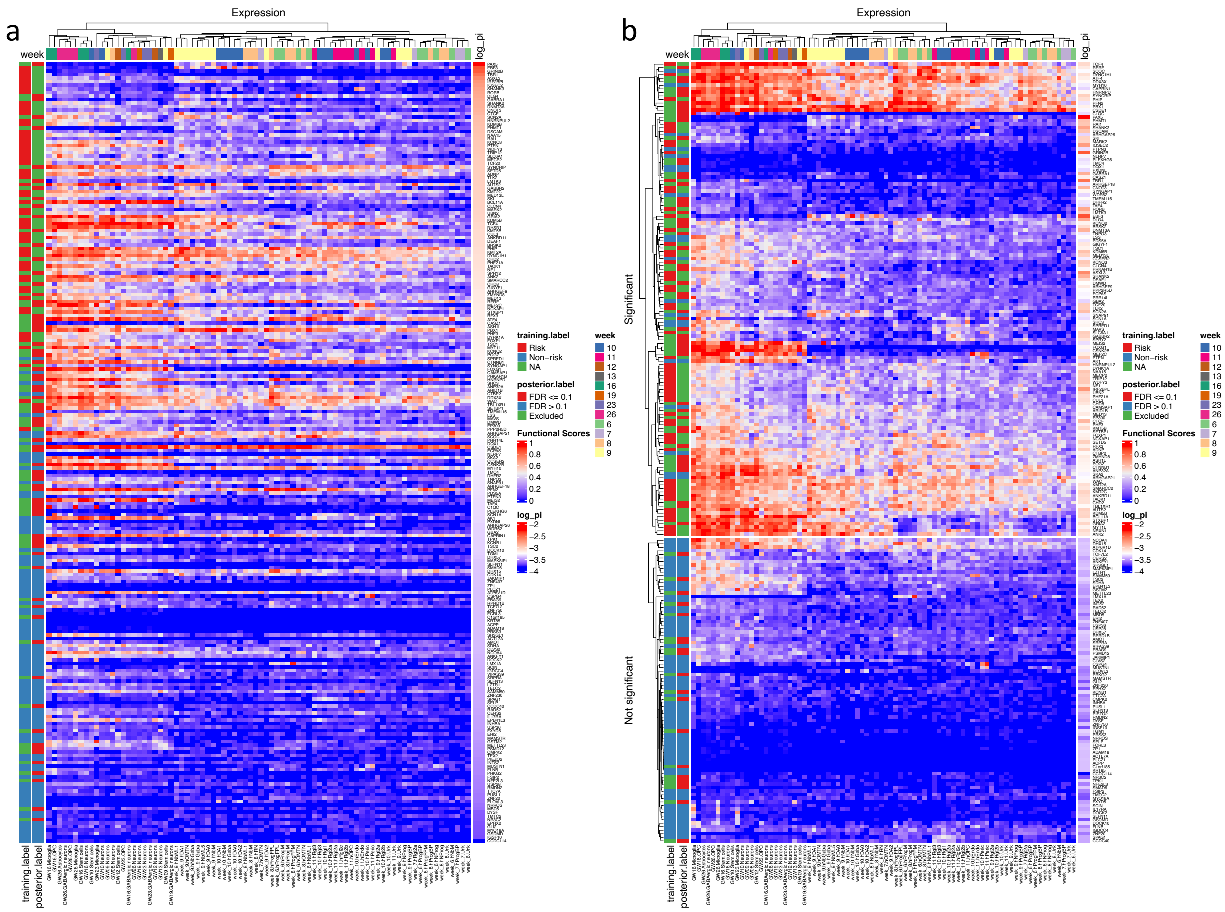

Supplementary Figure 4. Heatmap plot of gene expression profiles for some selected genes. We color the genes used in semi-supervised training as red (known disease risk) and blue (known disease non-risk) on the left, correspondingly (marked as “training.label”). We color the significant (FDR ≤ 0.1) genes and non-significant genes (FDR > 0.1) as red and blue (marked as “posterior.label”). We plot the log disease risk prior inferred by VBASS on the right (marked as “log_pi”). a) Rows ordered by log_pi. b) Rows ordered by hierarchical clustering as well as log_pi.

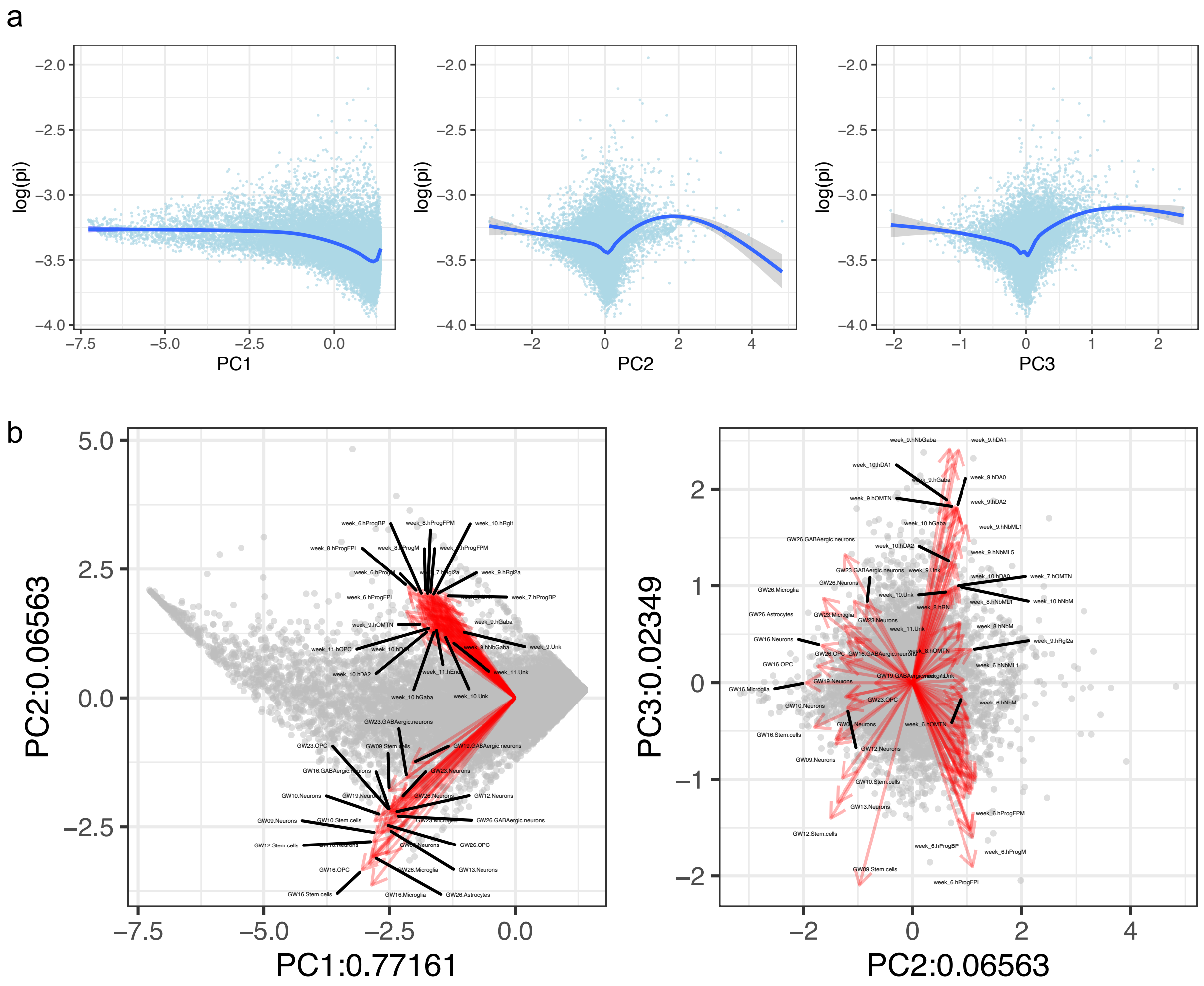

Supplementary Figure 5. The non-linear function between disease risk prior and cell type specific expressions. a) Visualization of the non-linear function of disease risk prior (log(pi)) on principle components (PCs) of gene expression. Each dot is a gene, x-axis showed its value of each PC, which is a linear combination of the co-expressed cell types, y-axis showed its log disease risk prior. The blue line showed a local regression line that approximately visualize the non-linear function. Grey shading showed the confidence interval of local regression estimated by geom_smooth function in R ggplot2 package. b) PCA plot of gene expression. Each dot is a gene, each arrow showed the importance of each cell type in that PC space. Here PC1 reflects whether the gene express or not, PC2 reflects the heterogeneity of the two datasets, PC3 reflects the heterogeneity of different neurons. From plot A and plot B we can qualitatively say that cells that have a large loading in PC1, PC2 and PC3, for example, GABAergic cells in week 9 (week_9.hNbGaba) will have a higher contribution to disease risk prior.

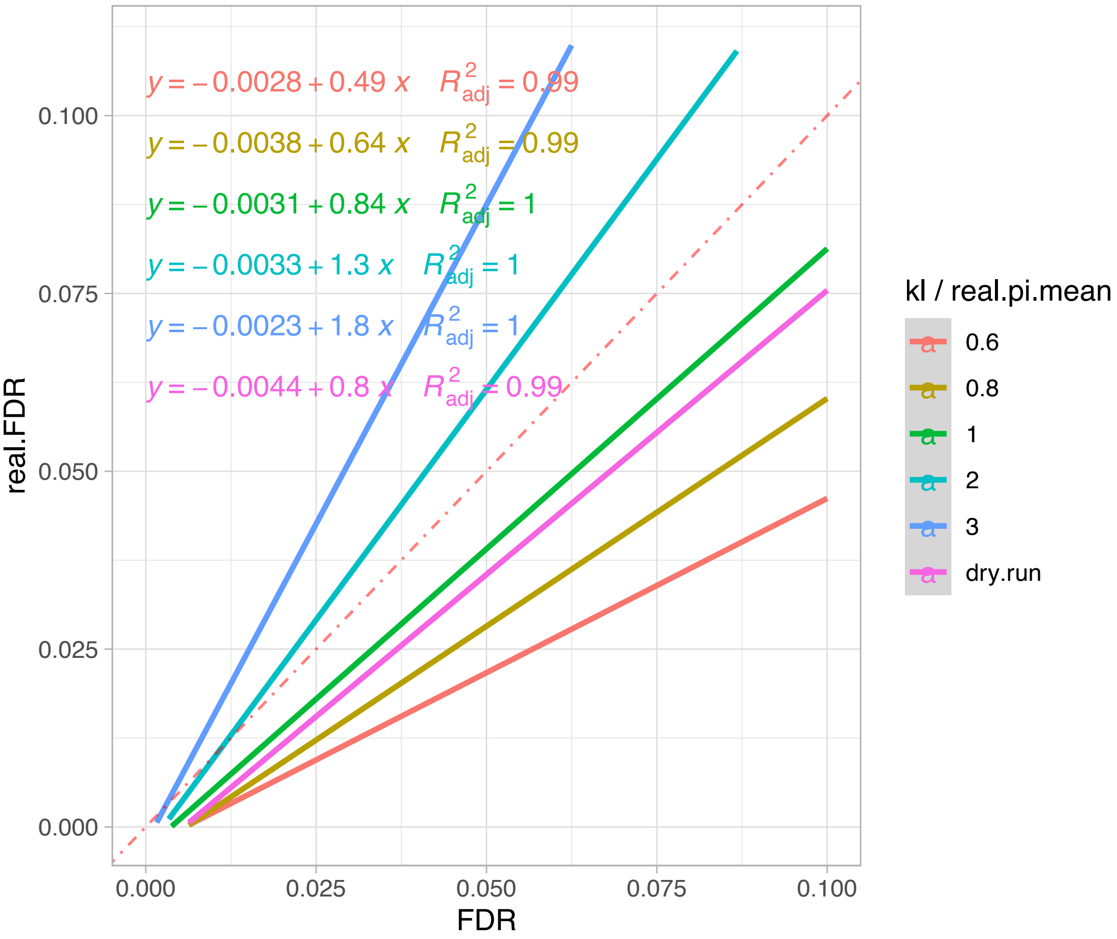

Supplementary Figure 6. The impact of KL penalty on false discovery control in simulation data. Each line represents the estimated false discovery rate (FDR, x-axis) and the real FDR (y-axis). We recommend setting the hyperparameter of KL penalty to the average of real disease risk prior, in other words, average proportion of disease risk genes, which can be inferred either from dry run of VBASS without expression data (pink) or from extTADA (green). Higher (cyan, blue) KL / real.pi ratio will result in improper false discovery control, lower (yellow, red) KL / real.pi ratio will result in stricter false discovery control and thus lower power.

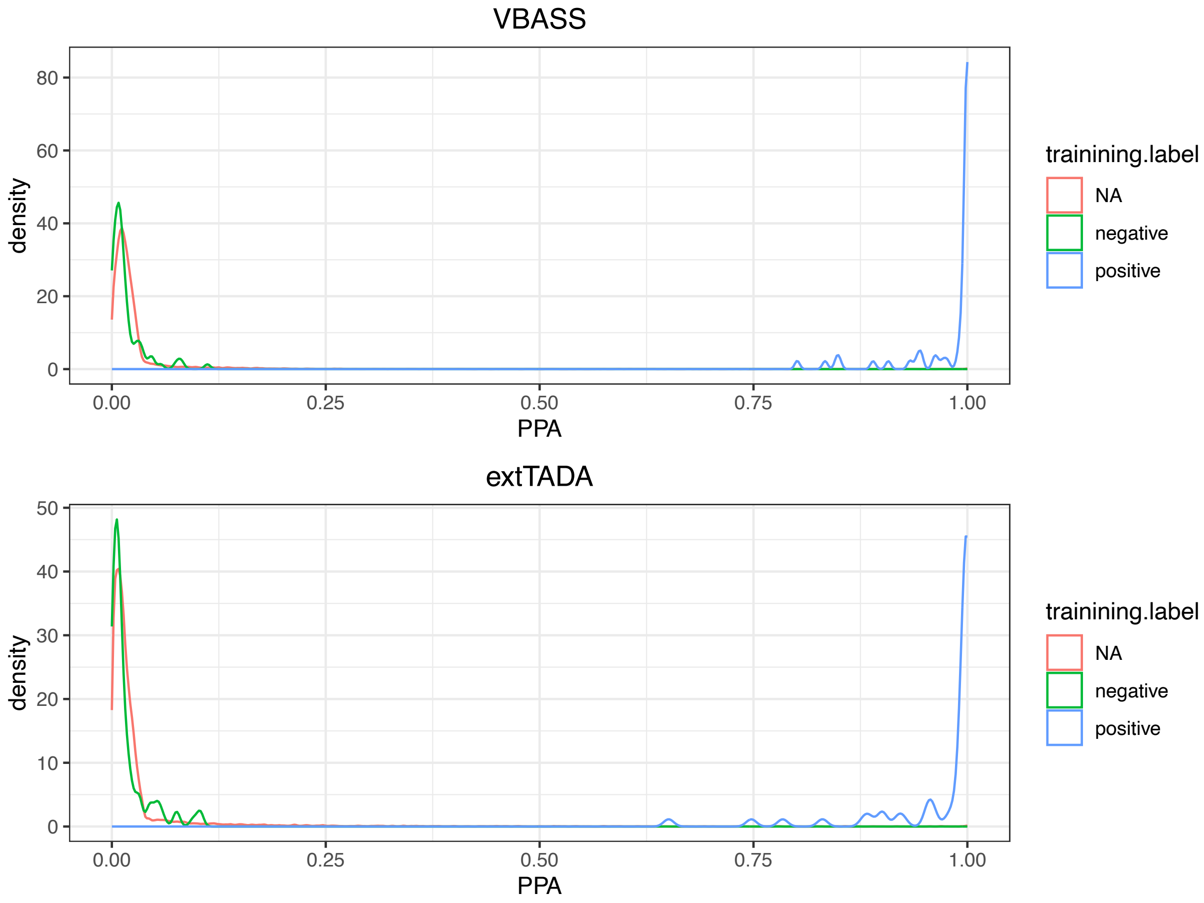

Supplementary Figure 7. Distribution of PPA value in VBASS and extTADA. Genes used in semi-supervised VBASS training were marked as blue and green, for positive and negative respectively. Other genes were marked as red.
