# Supplementary material for: VBASS enables integration of single cell gene expression data in Bayesian association analysis of rare variants
Source: Commun Biol. 2023 Jul 25;6:774. doi: 10.1038/s42003-023-05155-9 (PMC10368729; doi:10.1038/s42003-023-05155-9)
Supplement: Supplementary file 3 — Description of Additional Supplementary Files [file 42003_2023_5155_MOESM3_ESM.pdf]

<u>Description of Additional Supplementary Files</u>

File name: Supplementary Data 1.
Description: Labels of genes for VBASS in semi-supervised training.

File name: Supplementary Data 2.
Description: De novo variants of 2645 CHD trios in Jin et al 2017.

File name: Supplementary Data 3.
Description: De novo variants of 16616 ASD trios in Zhou et al 2021.

File name: Supplementary Data 4.
Description: Posterior probabilities of all genes calculated in CHD cohort by VBASS and extTADA.

File name: Supplementary Data 5.
Description: Posterior probabilities of all genes calculated in CHD cohort by VBASS and DECO.

File name: Supplementary Data 6.
Description: Posterior probabilities of all genes calculated in ASD cohort by VBASS and extTADA.

File name: Supplementary Data 7.
Description: Posterior probabilities of all genes calculated in ASD cohort by VBASS and extTADA. Removed positive training genes when calculating FDR.

File name: Supplementary Data 8.
Description: Posterior probabilities of all genes calculated in ASD cohort by VBASS and DECO. Removed positive training genes when calculating FDR.

File name: Supplementary Data 9.
Description: Source data sets for figures and supplementary figures.
